# A review of the implementation and research strategies of advance care planning in nursing homes

**DOI:** 10.1186/s12877-016-0179-4

**Published:** 2016-01-21

**Authors:** E. Flo, B. S. Husebo, P. Bruusgaard, E. Gjerberg, L. Thoresen, L. Lillemoen, R. Pedersen

**Affiliations:** Centre for Elderly-and Nursing Home Medicine, Department of Global Public Health and Primary Care, University of Bergen, P.O. Box: 7200, Bergen, Norway; Centre for Medical Ethics, Institute of Health and Society, University of Oslo, Oslo, Norway

**Keywords:** Advance care planning, Nursing home, Dementia, End-of-life care, Implementation, Barriers, Ethical decision making

## Abstract

**Background:**

Nursing home (NH) patients have complex health problems, disabilities and needs for Advance Care Planning (ACP). The implementation of ACP in NHs is a neglected research topic, yet it may optimize the intervention efficacy, or provide explanations for low efficacy. This scoping review investigates methods, design and outcomes and the implementation of ACP (i.e., themes and guiding questions, setting, facilitators, implementers, and promoters/barriers).

**Methods:**

A systematic search using ACP MESH terms and keywords was conducted in CINAHL, Medline, PsychINFO, Embase and Cochrane libraries. We excluded studies on home-dwelling and hospital patients, including only specific diagnoses and/or chart-based interventions without conversations.

**Results:**

Sixteen papers were included. There were large variations in definitions and content of ACP, study design, implementation strategies and outcomes. Often, the ACP intervention or implementation processes were not described in detail. Few studies included patients lacking decision-making capacity, despite the fact that this group is significantly present in most NHs. The chief ACP implementation strategy was education of staff. Among others, ACP improved documentation of and adherence to preferences. Important implementation barriers were non-attending NH physicians, legal challenges and reluctance to participate among personnel and relatives.

**Conclusion:**

ACP intervention studies in NHs are few and heterogeneous. Variation in ACP definitions may be related to cultural and legal differences. This variation, along with sparse information about procedures, makes it difficult to collate and compare research results. Essential implementation considerations relate to the involvement and education of nurses, physicians and leaders.

## Background

In modern Western society, an increasing number of individuals die from chronic debilitating conditions [1]. Death has been institutionalized; recent figures show that approximately 50–80 % of deaths in Europe occur during institutional stay and long-term care [2]. This is also the case in Norway, where almost half of the population dies in a nursing home (NH). Consequently, end-of-life care and treatment has been the object of increased interest in the primary health care system during the last few years [3].

To ensure that the period leading up to the end of a patient’s life is in accordance with the patient’s and family’s wishes, health personnel must guide patients and their family towards discussing and considering their current and future preferences pertaining to issues such as palliative care, symptom management, non-delayed dying process, spirituality and cultural setting. These themes are incorporated in Advance Care Planning (ACP), an ongoing communication and decision-making process with patients and relatives, addressing the approaching death and the practical challenges regarding ethics, treatment and care, well before the patient reaches a critical state [4, 5].

In Norwegian NHs, approximately 80 % of the long-term patients have mild to severe symptoms of dementia [6]. Patients suffering from dementia represent a special challenge, as they have often lost their ability to understand and make qualified statements and choices on their own. The optimal goal must be to openly discuss and document ethical and practical issues with the patients and their relatives before cognitive failure becomes a problem. Although death is often far from imminent when dementia is identified, the trajectory of dying is difficult to predict in this patient-group. Thus, correct and early timing in initiating ACP is of key importance [7, 8].

To meet the challenges of ensuring a dignified end-of life-period, written documentation (e.g., Advance Directives (AD)) of medical decisions relating to “do not resuscitate” orders, feeding tubes, and assisted respiration were originally promoted in these settings. However, this chart-based “tick off” system did not convey the patients’ underlying values nor did it stimulate individual discussions [9]. This distinguishes ACP from AD, as the latter focuses on clarifying treatment options of juridical significance by filling in a chart or legal form. As a result, the need for a more individual and flexible system became evident.

During the past decade, increasing interest in a multidisciplinary communication process with patients and relatives produced many different types of ACP-programs such as Let Me Talk (storytelling approach) [10], Let Me Decide [11], SUPPORT study [12], Respecting choices [13], Physician Orders for Life-Sustaining Treatment (POLST) [14], and Making Advance Care Planning a Priority (MAPP) [15]. Interestingly, the efficacy of ACP has been much debated, in part because it remains difficult to involve the participants’ family [10]. The use of ACP in a NH setting, especially with patients with dementia, remains a challenge that few ACP programs have been adapted to or tested for.

When assessing the efficacy of ACP, it is crucial to consider whether or not the ACP intervention has been properly implemented. Even if an intervention is superbly designed, real-world contextual factors may prevent the intervention from being realized as intended [16]. The intervention may not be carried out, or it may be conducted differently than intended. In other words, it is necessary not only to evaluate the effect of the intervention (e.g., reduced hospital admissions or more satisfied relatives) but also to evaluate implementation fidelity. Successful implementation may be challenged when the intervention is not experienced as relevant, workable or feasible. Implementation is still a somewhat neglected field of research, but it may maximize the impact of an intervention, or at least provide explanations for low efficacy [17]. Hence, in this review, we aim to emphasize the importance of implementation research when investigating complex interventions like ACP.

### Recent literature overviews of ACP

Various reviews have been conducted focusing on different issues related to ACP. A recent review by Fosse et al. [18], reviewed qualitative research investigating how physicians can improve end-of-life care (EoLC) in NHs. This review concluded that NH physicians were expected to comply with preferences for care, while at the same time providing guidance. The authors emphasize the need for physicians to recognize illness trajectories, and provide individualized ACP [18].

Another recent review investigated the effect of ACP on EoLC [19]. The authors concluded that ACP improved the quality of EoLC and suggest that complex and process-oriented interventions were more effective than chart-based interventions.

Van der Steen et al. identified aspects of the initiation of ACP in patients with dementia [20]. The authors found that most publications revolved around family issues, that is, their willingness or lack thereof, to start such a conversation. This review concludes that health personnel should initiate ACP early, yet be sensitive in terms of timing and approachability [20]. This review also emphasizes the complexity of ACP, and suggests that a simplistic chart-based approach should be avoided.

In a recent meta-analysis, Houben et al. investigated the efficacy of ACP interventions in different patient groups [21]. The authors found that the ACP interventions increase the completion of ADs and number of EoLC discussions, as well as enhance concordance between patient preferences and provided care [21].

Yet another review highlighted the gap between the number of elderly wishing to discuss their EoLC preferences, and the few who are actually given this opportunity [8]. The authors also highlight the fact that the end-of-life process may be unpredictable, and that a need for flexibility is not necessarily incorporated in an AD.

Robinson et al. investigated the effectiveness of ACP interventions in people with cognitive impairment and dementia. Interestingly, the authors conclude that it may be too late, in terms of decision-making capacity, to initiate ACP discussions when people with dementia are admitted to the NH [22].

Whereas most of these reviews rightfully underline the complexity of a successful ACP intervention, no review has made an in-depth investigation of the process and strategies of implementation. Details regarding implementation include whether or not personnel were trained, how other information was disseminated, and what barriers and promoters were operative in this process. In addition, these reviews have not focused on challenges that are unique to the NH setting, relating to implementation of a complex medical communication process in facilities with few physicians and a high number of patients with dementia. Thereby, the aim of this review was to investigate existing research that evaluates the implementation of an ACP intervention in NHs.

## Method

This scoping review of the literature aims to outline the process of implementation of ACP-related communication and end-of-life conversations discussing care and treatment with patients and relatives. This review has a specific focus on research and implementation strategies such as education and follow-up of staff, promoters and barriers. With this aim as our point of departure, we formulated the following research questions:

1. What was the content of the ACP interventions?
2. What ACP implementation strategies (training and target groups) were used and how were they described?
3. What were the main outcomes of ACP interventions in NHs?
4. What study designs and methods were employed?
5. What were the barriers and promoters of ACP implementation in NHs?

### Literature search

**Fig. 1:**
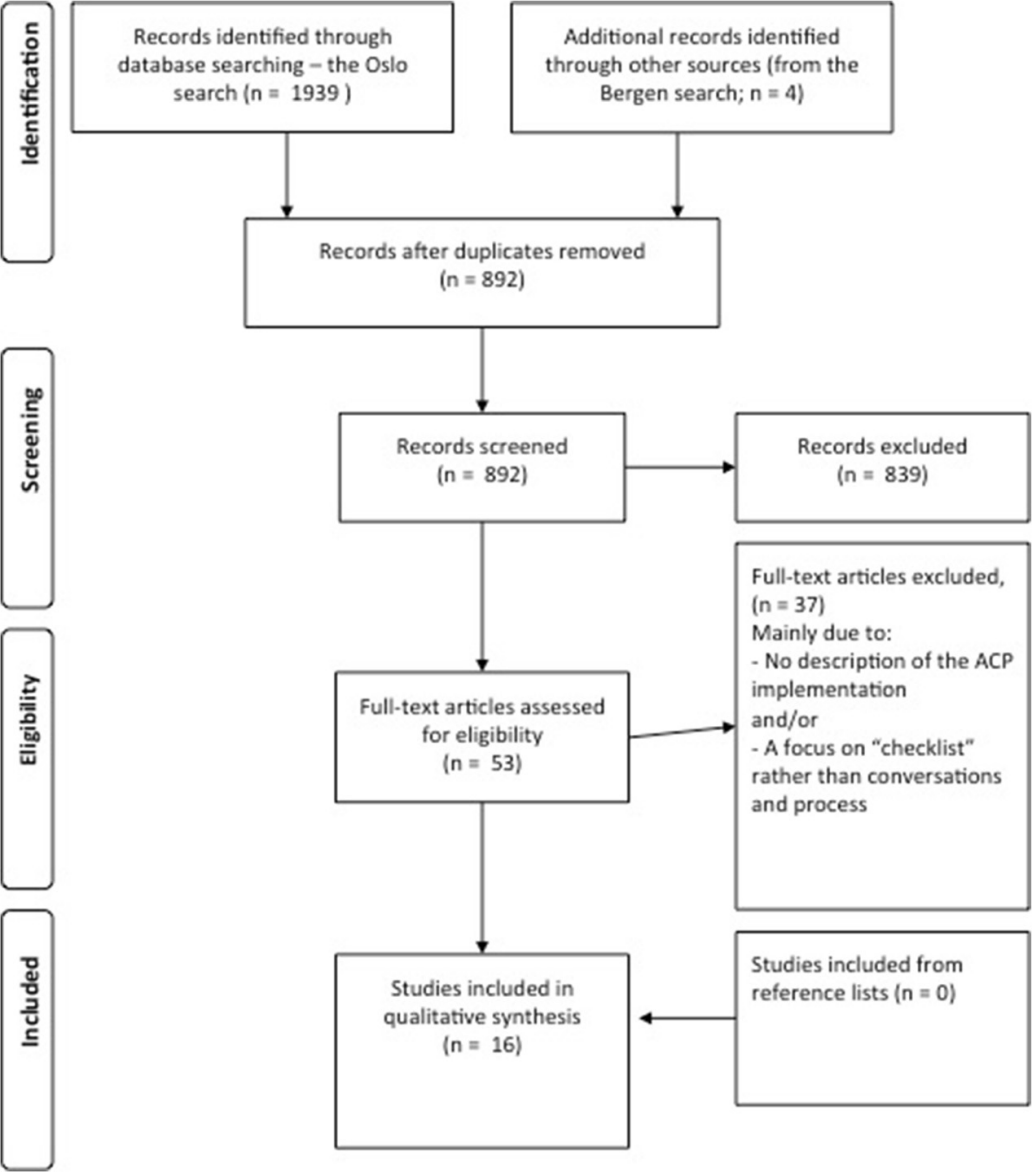
PRISMA based flow diagram of the review process

PICO-based searches (problem/population (P), intervention (I), comparison (C) and outcomes (O)) were conducted in January 2014 covering publications (original papers and systematic reviews) of research in medicine and social science (see Table 1 for a description of inclusion and exclusion criteria). To ensure that we identified all the relevant studies, two different research groups at the Universities of Bergen and Oslo performed two separate systematic literature searches, assisted by the university libraries in Bergen and Oslo, respectively. Keywords included MESH terms and phrases synonymous with “nursing home” and “advance care planning” (A complete overview of the different MESH terms and variable text that was used in the different databases are shown in Appendix Table 6). We searched CINAHL, Medline, PsychINFO, Embase and Cochrane libraries. In addition, we performed manual searches of reference lists in relevant publications (Fig. 1). It was challenging to identify a comprehensive set of keywords covering ACP-like interventions that were named before the MESH terms came into use in 2003; thus, we also included the older term “Advance Directives (AD)” in our search. The literature searches were then collated, and all authors discussed inclusion of publications.

**Table 1:** PICO-model of this systematic literature review’s research questions, inclusion and exclusion criteria

Population	NH patients and their relatives.
Intervention	Advance Care Planning defined as a conversation between patients, and/or relatives and health personnel about thoughts, expectations and preferences for end-of-life-care.
Comparison	All studies using standard care group comparison, before/after comparison, as well as studies without standard means of comparisons were included.
Outcome	All outcomes both qualitative and quantitative were included.
Exclusion criteria	Studies only including home-dwelling and hospital patients
	Studies only including specific diagnoses (e.g., heart failure, cancer)
	Studies only using chart based interventions where patients/relatives are left on their own (e.g., advance directives without conversations).
	Studies that only focused on treatment limits (e.g., DNR, DNH). Publications such as case studies, chronicles, guidelines, protocols, unsystematic reviews and legal documents were excluded.
	Publications in in other languages than English and Scandinavian.
	Publications without abstracts.

We included studies both with NH patients, their relatives and/or NH personnel as participants. Included studies used an ACP defined as a conversation between patients, and/or relatives and health personnel about thoughts, expectations and preferences for end-of-life-care. Studies using standard care group comparison, before/after comparison, as well as studies without standard means of comparisons were included. Both qualitative and quantitative study designs were included. No time limit were set. We excluded studies:

i) only including home-dwelling and/or hospital patients
ii) only including specific diagnoses (e.g., heart failure, cancer)
iii) only using chart based interventions where patients/relatives are left on their own (e.g., AD without conversations)
iv) only focused on treatment limits (e.g., DNR, DNH)
v) publications such as case studies, chronicles, guidelines, protocols, unsystematic reviews and legal documents and publications without abstracts.

Based on these exclusion criteria, all authors screened potential manuscripts at abstract level, and engaged in group discussions regarding all manuscripts read in full text and borderline exclusion cases (see flow chart over exclusion process, Fig. 1). After exclusion at abstract level, the review protocol of described inclusion and exclusion criteria was applied on selected full-texts (Fig. 1). We then searched through the reference lists of the included publications. We recognized that our specific search foci would yield few studies with a rigorous trial design. However, our goal was to identify studies on the process of implementing ACP in a NH setting. We did not endeavour to perform a meta-analysis of quantitative outcomes, but rather perform a scoping review, including several different study designs. Thus to rate the study methods and design according to one global quality checklist was beyond the frame of this review.

In order to extract and synthesize the content of the studies included in this review, manuscripts were read and discussed in groups. We agreed on the content to be extracted, which were then organized in a data-extraction table. The table were piloted, and discussed in the group. For each included study, we extracted the following information: full manuscript reference, number of participants, study design and method, type of intervention and control condition (if applicable), implementation strategy (including education) time to follow-up, study setting and outcomes. After agreeing on the format of data extraction, at least two of the co-authors read through the text independently and then verified the data-extraction in a discussion. Any unclear material was raised in group meetings. All authors partook in this process. Subsequently the organizing themes listed in Tables 2, 3 and 4 were formed in group discussions.

**Table 2:** Clinical intervention studies

Author	Population	Intervention-tool/education/aim of the study?	Comparison Methods Outcome measures	Outcome/themes/results	Promoters	Barriers
Livingston G, 2013 London, UK	Patients w/dementia who died before (*N* = 98), during (*N* = 56) or after (*N* = 42) the intervention mean MMSE = 5	Tool - GSFCH - Chart for choices Education - Learning course - 10-session manualized, interactive staff-training program Practical training - Facilitators	- Non-randomized intervention study, 2-year follow-up - Mixed methods - Interviews w/relatives - Review of med. records - QoL-AD, GHQ, DNR, ACP, days in hospital	- Better palliative approach - Fewer deaths in hospitals (from 76 to 47 %) - Better documentation of DNR orders (from 14 to 73 %) & ACP discussions (from 39 to 65 %) - No difference for days spent in hospital - More satisfied relatives - Staff more comfortable with addressing ACP-issues	- Staff training to increase awareness & knowledge & reduce fear - Motivated NH management - Trained in Gold Standard Framework - Low staff turn over	- Different dementia policy actions at the same time-change findings - Different cultures? Laws (e.g., Jewish tradition NH) - Adaption addressing different cultures in NHs necessary
Silvester W, 2013 Victoria area, Australia	19 Residential Aged Care Facilities (RACF) 203 Patients’ records Cognitive function not specified	Tool Making Health Choices	- Non-randomized controlled trial - Quantitative methods - Analysis of patient records, documented ACP pre/post-intervention timeframe not specified	- Better documentation of EOLC preferences & ACP discussions - 49 % MEPOA - >90 % value/beliefs - 78 % health perspectives	- Standards guiding ACP content & documentation - Ex. of values/belief statements in care plans - 17 principles of ACP (e.g., policies, education, information, routines, best interest, Inevitability of death, options, GP, EOLC, documentation confidentiality)	- Inconsistencies in naming & layout of ACP documentation
Hockley J, 2010, Scotland, UK	7 NHs 133 patients assessed as in need of ACP, who died during intervention, 95 controls (patients who died a year prior to intervention) 66 % were diagnosed with dementia	Tool - GSFCH - LCP Education - Learning course - Practical training - Workshops - Train the trainer - Facilitators - Support from researchers	- Intervention study, 18-months. follow-up - Mixed methods - Chart review - Survey of health care personnel - Qualitative interview of bereaved relatives (results not reported)	- Better palliative approach - Fewer hospital deaths - Staff comfortable with addressing ACP-issues	- Good consistent leadership - Regular visits from the same GP - More comprehensive palliative care approach	- Problems with staff turnover, retention & recruitment
Chan HY, 2010 Hong Kong	Competent NH patients: - 59 intervention - 62 control	Tool - Let me Talk Education - Semi-structured interview guide	- Non-randomized controlled feasibility study, 12-months. follow-up - Quantitative methods - Questionnaire based survey	- Only 3 families included - Stability of treatment preference - More preference stated - Relieved existential anxiety/distress		- Time consuming - Unclear effect in incompetent people/with dementia & older people
Morrison RS, 2005 NY City, USA,	- 4 Social workers (2 control/intervention) - 139 LTC residents: 96 control 43 intervention	Tool - Structured ACP discussion with patient & relatives at admission, 1 year & changes in clinical status Education - Counselling of NH social workers - Education/training: Terms/definitions, role-play, supervision - Practical training - Workshops	- Controlled clinical trial, 6-months. follow-up - Mixed methods - Minimum data set at admission - Interview of Social workers - Review of medical records	- Better documentation of EOLC preferences & ACP discussions - Better concordance between patient wishes & provided treatment	- High focus on decision capacity & proxy relative - Simple intervention of forms, team meetings, feedback to clinicians by social workers improves likelihood of residents preferences being elicited	- Few social workers - Lack of documentation - Short follow up - Legislation restricting surrogate decision making on behalf persons with reduced decision capacity

*ACP* advance care plan(ning), *EOLC* end of life care, *GSFCH* gold standards framework for care homes, *LCP* liverpool care pathway, *MEPOA* medical enduring power of attorney, QoL-AD, GHQ, DNR, ACP

**Table 3:** ACP tools with a chart-based focus, or Advance directive as main goal

Author	Population	Intervention-tool/education/aim of the study?	Comparison Methods Outcome measures	Outcome/themes/results	Promoters	Barriers
Hickman SE, 2011 Oregon, Wisconsin & West Virginia, USA	- 90 NHs - 870 Living & deceased residents with a valid POLST	Tool: POLST	- Cross-sectional observational study - Quantitative methods - Retrospective chart review	- Treatment for patients with a completed POLST mostly consistent with stated wishes: - Over 90 % adherence in terms of resuscitation, hospitalization & antibiotics, 63.6 % in terms of feeding tubes	Standardized medical orders that transfer with them throughout the healthcare system	
Sankaran S, 2010 Aukland, New Zealand	- NH & hospital nurses - Mental status not provided	- Multi-component support w/5main components: medication review, tel. hotline, advance nursing support POAC/Chronic Care Management programme & ACP Education - Learning course - Weekly in-house education - Practical training - Facilitators	Intervention study 6-months. follow-up - Mixed method - Observation & analyses of field notes. - Semi-structured interviews with staff pre/post intervention - Recording of medication changes, use of emergency calls & transmission to hospital	- No ACP were completed - All nurses but no physicians participated in the ACP-training - ACP programme continued - Education programme stopped	- Hotline - Education	- Unclear legal issues - Illnesses in the residents - Absent physicians - Staff was reluctance - Lack of time - Management thought residents’ cognitive state was too poor - The residents were insecure, as their family was not invited to the discussion.
Caplan GA, 2006 Australia	- 1 clinical nurse consultant - 2 hospitals, & 1 control hospital - 21 NHs - 45 NH patients - MMSE ≥16 excluded	Tool - “Let Me Decide” Education - Learning course - Education of family residents & staff about dementia, ACP, alternatives to hospitalisation - Facilitators	- Non-randomised intervention study, 12-months. follow-up - Quantitative methods - Controlled retrospective & prospective registry analyses over 3 years	- Changed routines, culture, - More information to families - Fewer deaths in hospitals - Decreased emergency calls in intervention hospital -Staff more confident in addressing ACP-issues	- Clarified role of the substitute consent giver - Capacity screening for mental competence by MMSE ≥16 - Education	- Challenges relating to following groups: dementia/neurodegenerative, cardiac & respiratory end-stage disease - Reluctance to sign the ACD document
Jeong SY, 2007 Australia	- 3 Patients - 11 Relatives - 13 Nurses - Final included N not specified	Not specified	- 7-months. observation study - Mixed method - Medical record analyses - Observation of specialist nurses & their role in the ACP process - Observation: residents, relatives & nurses - Interviews of staff, patients & relatives	Themes: - Nurses needed to clarify what ACP did & did not entail (i.e., dispelling myths such as ACP = euthanasia) - Nurses had an important role as a communicative link between physicians, family & patient		
Molloy DW, 2000 Ontario, USA	1292 Competent NH patients (MMSE > 16)/relatives of non-competent patients (Intervention *N* = 636, control *N* = 656)	Tool - Let Me Decide Education - Learning course - Practical training - Workshops - Train the trainer - Facilitators	Randomized controlled trial, follow-up at 6, 12 & 18 months. Quantitative methods Questionnaires to patients or patients relatives	- 49 % of residents & 78 % of relatives completed AD in intervention - Fewer hospitalizations - Reduced hospital costs	- Allocating personnel to ensure implementation	- The form was too comprehensive; deterred residents from completing it
Markson 1994	48 Competent NH patients 356 Home care patients 10 NH or home care Physicians			90 % of NH patients completed form		

*POLST* physician orders for life-sustaining treatment, *POAC* primary options for acute care

**Table 4:** Overview of process papers

Author	Population	Aim of the study?	Comparison Methods Outcome measures	Outcome/themes/results	Promoters	Barriers
Burgess M, 2011, USA	- 9 NHs - 31 physicians - 12 nurse practitioners/physician assistants	- Identify important barriers & promoters for ACP among NH staff	- Quantitative methods - Survey	- ACP documentation habits, i.e., location & who is responsible for documenting, perceived barriers & promoters - Experiences with different ACP elements	- Standardized form - Standardized location for documentation - Training/education of staff	- Patients’ impaired cognition - Lack of time during visit - Lack of family involvement
Stewart F, 2011 London, UK	- 34 NHs - 33 NH managers - 18 NH nurses - 10 Nurses & 29 care assistants from community - 15 Primary contact, family/friends - 14 Residents		- Qualitative Study - Semi-structured interviews about end-of-life care with staff & family members	Themes: - Benefits: choice, better planning, respect for patients wishes, aiding treatment decisions - Staff reported to have some form of ACP in place - Only 1 resident shared preferences, therefore interviews not included - Family & staff have different views about residents best interests	- Staff & family positive towards ACP; prepare for better planning - Early initiation; often too late in a NH - Family involvement - Familiarity between staff, resident & family - Staff training - ACP providing guidance to staff how to approach discussion	- Reluctant patients - Reluctant personnel, - Reluctant family involvement - Dementia - Unforeseen medical circumstances - Staffs diff. cultural beliefs, ethnic backgrounds - Family insists on hospital transfer - GPs not included-should be more engaged.
Froggatt K, 2009 UK	- 213 care home managers - 15 care home managers interviews	To describe current ACP practice in UK	Mixed method design in two cross-sectional phases - Questionnaire-based survey of 213 managers - Telephone based in-depth interviews	- 1/3 of the NHs had completed ACP in fewer than 25 % of the patients - 1/5 of the NHs had ACP completion in 75 % or more of the patients - 5 themes: consultation w/resident, consultation w/relative, discussing future decision making, training, manager perspective on ACP	- UK is engaged in strategy & policy initiatives for coordination of ACP - ACP Initiatives must consider implementation in which the whole system has to be considered	- Resident’s unwillingness & level of functioning, - Family unwillingness/availability/dynamic, - Staff confidence/knowledge/time/discomfort - NH resources - Extrinsic factors, i.e., GPs, district nurse & hospitals - Unclear responsibility
Shanley C, 2009 South Western Sydney, Australia	41 Care facility managers	To gain an understanding of how ACP is understood & approached by care facilities managers	Qualitative Study Interviews with managers Themes discussed: Initiation; Scope; Follow-up; Documentation; Organisational leadership; “In a nutshell” (individual initiative)	- Facilities without a systematic ACP approach tend to discuss EoLC late in illness - Little coherence between wishes & treatment plan - Common practice to incorporate ACP in the general care process - Conflicting ideas of ideal timing to initialize ACP	- Involve all stakeholders, - Systematic approach (i.e., guidelines, policies, protocols, checklists) - Clarified responsibility & documentation - Early initiation of ACP	- Patient & family unwillingness - Physicians’ reluctance - Legal uncertainties - Lack of training - No ACP system
Pauls MA, 2001 Toronto, Canada	7 nurses from Emergency Department (ED), 7 ED physicians 10 Paramedics 7 Long term care (LTC) nurses 4 LTC physicians	- Describe an ideal model for the transfer of an directive from LTC facilities to EDs - Understand the complex process in a transfer form	Qualitative study - 6 Focus group interviews with 35 participants	Theme –synthesis of the “ideal” ACP model: - Form: max 2 pp, simple language, specified options & room for alternative responses, physician’s signature - Completing the form: Education for staff, patient & family, starting early, process rather than a decision focus, yearly review, - Using the form: before acute illness, accessible, implement on regional basis, endorsed by authorities, improve staff education/communication	- Simplicity & acceptability - Physicians signature - Substitute decision maker - Education & repeated, simple info to patients & relatives - Process rather than a decision focus - Info in form of books, video, discussions - Cultural sensitivity	- In crises, physicians may not follow ADs/wishes - Minorities less likely to complete; mistrust - Unknown patients - Lack of time - Exclusion of physicians - Lack of external validity - Time consuming

*ACP* advance care plan(ning), *AD* advance directive, *ED* emergency department, *EoLC* end of life care, *LTC* long term care

## Results

In accordance to the Preferred Reporting Items for Systematic Reviews and Meta-Analyses (PRISMA), our search strategy is disclosed in the PRISMA- based flow diagram (Fig. 1). The systematic search generated 892 unique hits from both the searches in Bergen and Oslo. After exclusion at abstract level, the review protocol was applied on 53 full-text papers resulting in 16 included papers (Fig. 1). A search through the reference lists of the 16 included publications yielded no further publications.

The majority of studies employed different programs and interventions. The most frequently used ACP implementation strategy was staff education (learning courses and practical training). Effective implementation was reported to improve NH routines, culture, documentation of preferences, adherence to such documents, and fewer admissions and deaths in hospitals. Important promoters were education of staff, sufficient information on ACP, and standardization of ACP. Main barriers were absence of physicians, reluctance to initiate and participate in ACP discussions (personnel and relatives) and legal issues.

Although there were no geographic criteria, all but one paper were from English speaking countries. The included studies were situated in the USA, Canada, UK, Hong Kong, New Zealand and Australia. Both research teams used a data extraction sheet to collect information by the selected articles; then collected data were compared, double information removed, disagreements discussed, and agreement found for remaining data. We used the PICO model to organize and summarize the content of the included studies (Tables 2, 3 and 4). We included studies that aimed to implement ACP as a clinical intervention (Table 2), studies where the intervention was more chart-based, that is, aimed to complete AD forms (Table 3), and studies that mainly focused on understanding the ACP process (Table 4). In the first category, clinical interventions (Table 2), studies endeavoured to implement ACP in a clinical population of NH patients. This was also mainly the case in the second category; the chart-based studies (Table 3). Our goal was to include studies in which a communication process about preferences and values of NH patients were initiated. The chart-based studies were included due to this communication focus, even though they treat the completion of charts an important outcome. The last category (Table 4) included studies evaluating the process of implementing ACP, providing in-depth information on typical barriers and promoters encountered throughout implementation. Akin to this, the studies summarized in Table 4 may be described as employing a formative evaluation in which the researchers and the informants sought to recognize and respond to the barriers and promoters of ACP, and thereby to enhance implementation.

### What was the content of the ACP interventions?

As described in Tables 2 and 3, most studies employed different ACP interventions. The only overlapping use of ACP tools were evident in Caplan et al., and Molloy et al., who both employed “Let Me Decide”, and Hockley et al. and Livingston et al., who used the gold standards framework for care homes (GSFCH). For a closer description of the different ACP tools, please see Table 5.

**Table 5:** Description of ACP Tools employed in studies included in the review

Physician Orders for Life Sustaining Treatment (POLST), Hickman [28]	The POLST is collected through conversations between patients, relatives, and health personnel about preferences for EoLC. It is form-based and designed to function as a directive for treatment, covering issues like A-C: CPR, medical intervention, antibiotics and nutrition in case of any changes in a patient’s condition.
Gold standards frame-work for care homes (GSFCH), Hockley [24], Livingston [26]	The GSFCH is a quality improvement program with education modules that focus on ACP. The framework also aims to formalize the ACP using a form that includes open-ended questions about preferences for care and aims to determine whether a Lasting Power of Attorney is mentioned.
Let me talk, Chan & Pang [10]	Let me talk is based in four meetings sequentially covering the following themes: life stories, illness narratives, life views and end-of-life care preferences. A semi-structured interview guide assists the facilitating nurses. The sessions aims to accumulate in a personal booklet documenting the patient’s individual life stories, health care concerns, preferences for life-sustaining treatment and potential decision-maker
Let Me Decide, Caplan [27], Molloy [11]	This approach is based on conversations with patients and relatives, with the aim of completing a legally binding document which the “Let Me Decide: Health and Personal Care Directive” form is in Canada and Australia
Advance Directives Markson [25]	Here Advance Directives entailed in depth discussions between physicians, patient, and relatives, and would likely be defined as ACP today.
Making Health choices, Silvester [30]	Standardized contents of ACP discussions; should include in own words: Current health state, current goal, values & beliefs, future preferences; decision maker

Not all studies employed an ACP “standard” as listed in Table 5. Sankaran et al. had a complex intervention in which not all tools related directly to ACP. With this non-standard ACP framework, the documented preferences were neither legally nor clinically binding. Nurses initiated ACP without including relatives, and without the evaluation of medical status and prognosis by a physician [23]. Although the tools were diverse, ACP was by and large defined as a decision-making process. Meanwhile, there was variation between the studies in terms of how official or formalized the ACP conversation and documentation was. Some regarded the ACP as a means by which to obtain a directive, while others focus on the “good conversations”, being seen and heard and preparing for the inevitable.

### What ACP implementation strategies were used and how were they described?

To ensure the quality and implementation of the ACP, different educational approaches were employed (Tables 2 and 3). Some studies used a comprehensive strategy including learning course, practical training and facilitators who helped disseminate the training to other staff in the included NHs [11, 23–26]. Education as implementation strategy was not used in six of the included studies [10, 11, 27–30]. Molloy et al., Caplan et al., Sankaran et al., Morrison et al., Hockley et al. and Livingston et al. all used a multicomponent educational program including several sessions, multiple recipients (nurses, physician families), and both courses and practical training. Yet, none of the studies described in full the education content and form.

#### Target groups and study participants

A majority of 12 included studies focused on health personnel as study participants/informants; nine of these studies included NH staff [11, 23, 24, 26, 27, 29, 31–33]; three included NH physicians [25, 31, 33]; one included hospital physicians and paramedics [33]; three studies included facilitators in NHs [24] and three included managers [32, 34, 35]. In addition, Morrison et al. included NH social workers [36]. While nursing staff was the most frequent target group for training and education, some studies also offered training to physicians to initiate and support a formal ACP process [23, 27]. Noticeably, the inclusion of physicians proved more difficult. None of the NH physicians included in the study by Sankaran et al. actually participated in the education and ACP intervention. The authors noted that the patients found it difficult to make decisions without a medical review, suggesting that it was problematic that the physicians were not present to explain prognosis and options. The study does not describe the strategy used to include physicians in the study or the ACP discussions.

While mainly staff members were targeted for education, some studies included relatives to ascertain their perception of the ACP process [26, 29, 32]. Caplan et al. also focused on providing information and education to the relatives regarding the terminal nature of dementia, and the contents and goal with ACP [27]. Caplan et al. observed that most families had not previously been educated on the terminal nature of dementia. Family members were relieved to have this information, which allowed them to plan ahead. Few studies aimed to include patients as study informants and those who did, excluded patients with more advanced dementia. For example, Caplan and colleagues included patients 65 years or older who provided consent (*N* = 45) and employed the Mini Mental State Examination (MMSE) >16 as the cut-off for involving patients in education. Moreover, a large part of the studies did not implement ACP in those NH patients who had dementia. This excludes a large segment of the NH population. Indeed, Burgess [31] concluded that it was even more important to properly complete ACP for patients who are losing their ability to communicate their wishes [31]. Yet, this large patient-group remains neglected in research.

Other studies investigated how patients fared with an ACP intervention through medical records, that is, not including them as informants or active study participants. For example, Hockley and colleagues investigated medical records for residents, 66 % of whom were diagnosed with dementia. They investigated the presence and nature of ACP prior to the interventions (control group, *N* = 95), and while the intervention was implemented (intervention group *N* = 133). Both controls and intervention participants were included if they were assessed as needing ACP.

### What were the main outcomes of ACP interventions in NHs?

Many of the included publications focused on implementing ACP to successfully change NH routines and culture [27]. Studies reported an improved palliative care approach [24, 26], fewer deaths in hospitals [24, 26, 27], and reductions of hospital admissions with related costs [11, 31]. Burgess also found that ACP interventions led to reductions in invasive procedures [31]. Sankaran et al. also evaluated the appropriateness of hospital admissions as an outcome, but a poor implementation, that is, no completed ACP, led to inconclusive results [23].

Several studies had the completion and documentation of ACP discussions and medical decisions as their main aim and study outcome. A successful implementation of ACP was shown to yield better documentation of discussions and EoLC preferences [24, 26, 30, 36]. Markson and colleagues found that 65 % of residents who were approached by their physician for discussion made statements relating to treatment preferences [25]. Chan et al. also described an increased prevalence of documented preference [10]. When surveying the use of ACP in NHs, Froggatt et al. found that one in three participating NHs had provided ACP to fewer than 25 % of the patients/relatives, while in a fifth of the NHs, 75 % or more had received ACP [35]. Although many barriers were successfully identified in this study (Table 4), factors for success in the NHs who delivered ACP to ≥75 % were not specified.

Though many studies included the number of documented ACP discussions as an important study outcome, such documents may still not be considered in medical decision-making. Hickman and colleagues explored whether documented patient preferences were respected. A high correlation was found between the initial POLST orders and final treatment (93–98 % match in relation to different treatment options), with exception for use of feeding tubes (64 %) [28]. Morrison and colleagues found that ACP led to a better concordance between patient wishes and provided treatment [36] and similarly, Silvester and colleagues found a better adherence to the preferences documented through ACP [30].

Three studies found that the ACP intervention made staff more comfortable with addressing emotional needs and discussing issues relating to irreversible illness and death with patients and patient relatives [24, 26, 27]. Meanwhile, one study found that relatives wanted the documentation and communications relating to ACP to be provided by a physician [31].

Few studies had patient data as their main focus. Importantly, Burgess and Chan reported beneficial patient outcomes such as peacefulness [31], and eased existential distress [10]. Also relatives reported increased satisfaction with decisions [26].

### What study designs and methods were employed?

The method and design was often superficially described, making it difficult to assess the quality of the included publications. Few of the publications described the NHs and participants that were included in the study. In addition, there were no descriptions pertaining to how dropouts were managed and few described how the cognitive status and ability to give consent were evaluated in the NH patients. No study provided a power analyses. In addition, most studies employed an open (not blinded) study design. Taken together, the studies included in this review may have biases.

Five of the included studies investigated ACP as a clinical intervention (Table 2). Six studies investigated the use of ACP, yet with a focus on completing ADs or similar chart based approaches (Table 3). Five studies investigated the process of successfully implementing the use of ACP in NHs.

Five studies used a mixed methods approach [23, 24, 26, 29, 35]. All of these employed qualitative interviews to ascertain the experience of the ACP intervention. Three of these studies also used quantitative analyses in which events were registered and counted from field notes [23, 26, 29].

Three studies only performed qualitative interviews to investigate the ACP routines [32–34]. Five studies only employed a quantitative method of investigation [10, 27, 28, 30, 31].

### What were the barriers and promoters of ACP implementation in NHs?

In terms of barriers, eight studies identified challenges relating to relatives and/or patients, including reduced mental capacity [10, 23, 31, 32, 35] and unwillingness/reluctance to discuss the impending future and related ACP issues [23, 25, 27, 31, 32, 34, 35].

The majority of the studies identified barriers relating to health personnel and organizational issues. The health personnel were reluctant or ambivalent to discuss ACP related issues [23, 32, 34, 35].

Interestingly, several systems-related issues were identified, including lack of competence and experience [25], uncertainty about the legal implications of patient and family statements [25, 34], and resource problems (e.g., staff shortage, turnover, lack of time) [23–26, 31, 35]. Unconstructive culture and lack of administrative support were factors that were also identified as barriers [25, 32, 34].

In the study by Sankaran, et al., no ACP discussions were documented. The physicians did not use the ACP education that was offered to both nurses and physicians. In addition, the juridical questions that arose during the trial hindered implementation.

One study identified unforeseen medical scenarios as a barrier [32], while six studies did not describe barriers [11, 26, 28–30, 36].

Several promoters for ACP implementation were identified, of which “education” was most frequently listed as an important contributing enabler [11, 25–27, 29, 31–34, 36]. Similarly, providing information about ACP was highlighted in four of the publications [11, 29, 33, 34].

Several studies emphasized the importance of standardization; in terms of both the ACP form and process [30, 31, 33, 34], and where the ACP was documented [30, 31, 34].

In order to successfully implement a demanding intervention, one of which may arguably be ACP, the NH system needs to put its support behind the intervention. Not surprisingly, good and consistent management was identified as an important promoter in six of the included publications [23, 24, 26, 27, 29, 34]. Additionally, the physician was identified as an important agent; both as the one initiating the ACP discussion [25], and the fact that the same physician had regular visits [24]. Similarly, Burgess found that the relatives wanted the ACP to be provided by the physician.

The acquaintance between the health personnel, patient and their relatives was also highlighted [32], as was the involvement of family members [32, 33].

Two studies specifically addressed timing, concluding that an early intervention with follow-up discussions promoted a successful ACP intervention [32, 33].

## Discussion

In this review, we found 16 publications with heterogeneous study design and quality. The chief ACP implementation strategy was staff education (learning courses and practical training). Effective implementation successfully improved NH routines, culture, documentation of conversations and preferences and enhanced adherence to such documents, as well as fewer admissions and deaths in hospitals. Important promoters for successful implementation were education of staff (providing security and confidence), the provision of information regarding ACP, and standardization of the ACP process (responsibility, content and documentation). Main barriers for successful implementation were non-attending NH physicians, reluctance among both personnel and relatives to initiate and participate in ACP discussions and legal uncertainties. Although cultural and legal aspects were only sporadically mentioned in the included publications, they may have a major influence on ACP content, implementation, outcomes, methods and barriers and promoters [23, 37].

Most of the included studies highlighted that ACP is a process rather than “the one big talk”. However, we found that the ACP intervention varied greatly in content, scope and target groups. Some of the variations derive from the different definitions of ACP. While most chose to define ACP as a decision-making process, some emphasized the preparing of relatives and patients for the final days and the potential end-of-life trajectory [29]. The definition of ACP and the variation as to of how formalized the ACP conversation and documentation was seems closely intertwined with the legal considerations unique for each country where an ACP intervention has been implemented and investigated.

### Legal considerations

The legal mandate for decision-making, the legal implications of stating end-of-life wishes, and the need for a directive vary across the borders, both between nations and states. The various legal decrees dictate to some degree the urgency and the focus of an ACP discussion.

While 15 European countries have specific legislation relating to ADs, several countries (e.g., Ireland, Italy, Poland and Sweden) have not yet ratified such laws. Those countries with specific legislation for end-of-life decision-making and ADs vary regarding documentation, terms for validity of the document, the rights and responsibilities relating to durable power of attorney, and how widely used the law is [37].

In Norway, the physician has the final word in medical decisions, but all decisions should ideally be in accordance with both the patients’ preferences and the patient’s best interest (as evaluated by the physician). There is no official standard for the EoLC in Norwegian NHs, and the communication between staff and relatives represents a challenge [38]. In effect, the variation between both countries and institutions is vast; some patients are seldom forced to receive invasive treatment like feeding tubes [39], while in other instances, this clinical intervention is more common [40].

In the United States, the Patient Self-Determination Act mandates that federally funded health care organizations must advise patients of their right to make end-of-life decisions in advance. In Canada, a majority of provinces have legislation recognized ADs [33].

In the study by Sankaran and colleagues, the legal aspects in New Zealand were highlighted as an important barrier. Only the patient could make a plan for future personal care; if the patient was incompetent to make decisions, the New Zealand law did not permit an appointed person to make any statements on behalf of the patient. The introduction of ACP was delayed by the need for a legal review of the documents. During the six-month intervention, no ACPs were completed. Meanwhile, in Australia, an Enduring Power Attorney can complete, on behalf of the person, an ACP. Nevertheless, in one Australian study, the ACP uptake was low [27].

### Cultural aspects

ACP definitions and content varies from study to study. This may be due in part to the different national legal constraints, but it may also reflect cultural differences in terms of what the NH staff, the family and patients expect. What is considered to be a good and dignified death? What is needed in the NH setting? This may be illustrated in part by the introduction to the Chan et al. paper stating that family members and health care providers often want to protect the patients from sensitive issues, but highlighting the fact that “such a conspiracy of silence does not necessarily prevent older people from thinking about these issues” [10]. In the British study by Stewart, some participants reported a reluctance to initiate discussions and the need to commence gradually, while others in the same study considered a direct approach to be preferable. It is possible that in this multicultural world, inter-individual differences are just as great as differences between cultures and nations. Some need time to be able to discuss these difficult issues; others cannot wait to get their worries of their chest.

Not only do the definitions of ACP differ, but the definition, organization, and mandate of NHs vary between countries as well. In some countries like Norway or the Netherlands, NH care is a public service offered to those in need of it. In other countries, NHs are private institutions, in which admission is to a lesser degree justified by care needs. Thus, some NHs are mainly inhabited by very old and frail patients with dementia, while this is not necessarily the case in other countries. Such differences affect the form of the ACP. For example, in Norway, most patients admitted to a NH have various degrees of dementia, and many patients have lost the ability to make meaningful choices about future care before admission [41].

There seem to be slight but important cultural differences in terms of what a successful outcome of an ACP intervention is. Chan and colleagues highlighted the difficulty in involving relatives, and defined the participation of family members as an important outcome [10]. Another cultural aspect is evident in what is considered to be a failed process. In a paper by Forbes et al., letting some of the treatment decisions be taken by the clinical staff is indirectly described as a negative outcome; “Some, either actively or passively, allowed providers, nursing facility personnel, to become the decision makers” [42]. Here the importance of being autonomous decision makers may be a cultural value. Meanwhile in other countries it might be expected that the nurses and physicians take a more decisive role.

Several studies emphasize that the NH physician must take the initiative and accept the leading role as conductor in this process; otherwise, communication in the process will develop huge and unnecessary gaps and remain fragmentary [33]. On the other hand, studies demonstrated that nurses are invaluable facilitators in making the voices of patients and relatives heard, their values known, and their care preferences clarified [10, 11].

Most of the studies included in this review seem to share the idea that reductions in unnecessary treatment and hospitalization represent a positive outcome of ACP. Meanwhile, withholding treatment may be perceived as medical neglect and may even be misinterpreted as euthanasia if taken out of context and not communicated properly, as discussed by Jeung and colleagues. Although ACP is not euthanasia, which is illegal in most countries, the line between a reduction in “unnecessary” treatment and neglect, or therapeutic nihilism, may be thin or difficult to draw. Hence, it is vital to be constantly aware of the fact that a reduction in hospital admissions or invasive treatment is not always a sign of improved treatment and care [27].

How ACP is received may in part rest on how NH staff presents the goal of ACP. In addition, the ease of implementing the ACP intervention may be affected by whether or not there is a culture for ACP-like communication among healthcare personnel.

### Tools adapted for use with patients without consent?

It is discouraging that many ACP tools are not designed with patients with dementia in mind. Special attention should be given to ethical issues such as informed consent and presumed consent. The physician’s statements should be reflective and clear, especially regarding who is responsible for what, and they should invite questions and discussion. A summary of the meeting must be documented in the patient’s chart and made available to all personnel involved. During the next weeks and months, follow up meetings should be planned and organized, especially when life-threatening complications occur.

### Methodological issues

Few studies employed a blinded controlled trial design, there were generally few participants, and no study reported analyses of statistical power. Unfortunately, most of the publications did not sufficiently describe the inclusion of NHs and participants, management of dropouts, or how cognitive status and ability to consent were ascertained. The potential lack of power and selection bias, means that the studies included in this review may include methodological and statistical biases that have not been properly taken into account. Moreover, the implementation process, and the education provided were not described in detail. There was also a wide variation both in the interventions used and in the study designs.

Hence, a limitation with our literature review is restricted possibility to compare the studies in terms of quality and methods. Furthermore, conclusive recommendations based on aggregated evidence are nearly impossible to make, and this in turn limits the conclusions that are possible to be drawn based upon this review. Due to our choice to include both qualitative and quantitative studies, focusing on the implementation and process instead of outcomes, a meta-analysis was also beyond the scope of this current review. We acknowledge also that in this integrative focus were discussion of process was valued the use of standardized study quality grading systems were not used.

## Conclusion

Implementation and testing of research-based ACP in NHs and people with dementia remains an important challenge, there is still a need for well-powered randomized trials to investigate the efficacy of different interventions. This means that there is a need for high quality studies that describe in detail the ACP-process, the implementation strategies and the study design including robust primary and secondary outcome measures. For now, studies suggest that essential implementation considerations entail the involvement and education of staff, including nurses, physicians and NH leaders. Furthermore, researchers should consider how to balance the need for both outcome and process evaluation and how to include patients with cognitive impairment and their relatives in future ACP-studies in NHs.

## Appendix

**Table 6:** List of MESH terms and Free text search terms used in different databases

Database:	Ovid MEDLINE(R) In-Process & Other Non-Indexed Citations and Ovid MEDLINE(R) 1946 to Present
	Search terms Advance Care Planning:
	*MESH terms*
	Advance Care Planning
	Advance Directives
	*Free text*
	(advance* adj (care plan* or health care plan* or healthcare plan* or medical plan* or treatment plan* or directiv* or care directiv* or health care directiv* or healthcare directive* or treatment directiv* or care wish* or treatment wish*)).
	((advance adj3 plan*) or ((living or patient) adj2 (will* or contract* or decision* or participat*)) or (advance adj1 directive*) or (Attorney adj2 Power) or (psychiatric adj1 will*)).
	(end of life adj (decision* or communicat* or care communicat* or discussion* or plan* or care plan* or wish* or conversation*)). (plan* for the end of life or plan* for end of life).
	Search terms nursing home:
	*MESH terms*
	Homes for the aged
	Nursing homes
	Long-Term Care
	Hospices
	*Free text*
	(nursing home* or “home* for the aged” or hospice*).
	(nursing home* or care home* or long-term care or longterm care or old peoples home* or rest* home* or home* for the aged or intermediate care facilit* or skilled nursing facilit*).
	Search terms patient group – dementia:
	MESH terms
	Dementia
	Alzheimer disease
	Frontotemporal lobar degeneration
	Lewy body disease
	Delirium
	Amnestic
	Cognitive Disorders
	*Free text*
	(dement* or alzheimer* or “Frontotemporal lobar degeneration” or “Lewy Body disease”).
Database:	Embase (Ovid) 1947–2014
	Search terms Advance Care Planning:
	*MESH terms*
	Living will
	Patient decision making
	*Free text*
	(advance* adj (care plan* or health care plan* or healthcare plan* or medical plan* or treatment plan* or directiv* or care directiv* or health care directiv* or healthcare directive* or treatment directiv* or care wish* or treatment wish*)).
	((advance adj3 plan*) or ((living or patient) adj2 (will* or contract* or decision* or participat*)) or (advance adj1 directive*) or (Attorney adj2 Power) or (psychiatric adj1 will*)).
	(end of life adj (decision* or communicat* or care communicat* or discussion* or plan* or care plan* or wish* or conversation*)).
	(plan* for the end of life or plan* for end of life).
	Search terms nursing home:
	*MESH terms*
	Home for the aged
	Nursing home
	Long term care
	Health care facility
	Hospice
	*Free text*
	(nursing home* or “home* for the aged” or hospice* or care home* or long-term care or longterm care or old peoples home* or rest* home* or intermediate care facilit* or skilled nursing facilit*).
	Search terms patient group – dementia:
	*MESH terms*
	Dementia
	Alzheimer disease
	Diffuse lewy body disease
	Frontotemporal dementia
	Mixed depression and dementia
	Senile dementia
	*Free text*
	(Dement* or alzheimer* or “Frontotemporal lobar degeneration” or “Lewy Body disease”).
Database:	PsycINFO (Ovid) 1806 to October Week 2 2013
	Search terms Advance Care Planning:
	*MESH terms*
	Advance directives
	*Free text*
	((advance adj3 plan*) or ((living or patient) adj2 (will* or contract* or decision* or participat*)) or (advance adj1 directive*) or (Attorney adj2 Power) or (psychiatric adj1 will*)).
	advance* adj (care plan* or health care plan* or healthcare plan* or medical plan* or treatment plan* or directiv* or care directiv* or health care directiv* or healthcare directive* or treatment directiv* or care wish* or treatment wish*)).
	(end of life adj (decision* or communicat* or care communicat* or discussion* or plan* or care plan* or wish* or conversation*)).
	(plan* for the end of life or plan* for end of life).
	Search terms nursing home:
	*MESH terms*
	Residential care institutions
	Nursing homes
	Hospice
	Long term care
	*Free text*
	(nursing home* or care home* or long-term care or longterm care or old peoples home* or rest* home* or home* for the aged or intermediate care facilit* or skilled nursing facilit* or hospice*).
	Search terms patient group – dementia:
	*MESH terms*
	Dementia
	Dementia with lewy bodies
	Senile dementia
	Vascular dementia
	Alzheimer’s disease
	Senile plaques
	*Free text*
	(dement* or alzheimer* or “Frontotemporal lobar degeneration” or “Lewy Body disease”).
Database:	CINAHL - Search modes - Boolean/Phrase
	Search terms Advance Care Planning:
	*MESH terms*
	Decision Making, Patient
	Advance Directives
	Advance Care Planning
	*Free text*
	TI (((advance N3 plan*) or ((living or patient) N2 (will* or contract* or decision* or participat*)) or (advance N1 directive*) or (Attorney N2 Power) or (psychiatric N1 will*))) OR AB (((advance N3 plan*) or ((living or patient) N2 (will* or contract* or decision* or participat*)) or (advance N1 directive*) or (Attorney N2 Power) or (psychiatric N1 will*)).
	TX advance* N1(care plan* or health care plan* or healthcare plan* or medical plan* or treatment plan* or directiv* or care directiv* or health care directiv* or healthcare directive* or treatment directiv* or care wish* or treatment wish*)
	TX end of life N1(decision* or communicat* or care communicat* or discussion* or plan* or care plan* or wish* or conversation)
	TX plan* for the end of life or plan* for end of life
	Search terms nursing home:
	*MESH terms*
	Long Term Care
	Nursing Homes
	Nursing Home Patients
	Skilled Nursing Facilities
	Hospices
	Hospice Patients
	Free Text
	*Free text*
	TI ((nursing home* or “home* for the aged” or hospice*)) OR AB ((nursing home* or “home* for the aged” or hospice*))
	TX nursing home* or care home* or long-term care or longterm care or old peoples home* or rest* home* or home* for the aged or intermediate care facilit* or skilled nursing facilit*
	Search terms patient group – dementia:
	*MESH terms*
	Delirium
	Dementia
	Amnestic
	Cognitive Disorders
	Dementia
	Dementia, Senile+
	Lewy Body Disease
	Dementia, Multi-Infarct
	*Free text*
	TI ((dement* or alzheimer* or “Frontotemporal lobar degeneration” or “Lewy Body disease”)) OR AB ((dement* or alzheimer* or “Frontotemporal lobar degeneration” or “Lewy Body disease”))
Database:	Cochrane library
	Search terms Advance Care Planning:
	*MESH terms*
	Advance Care Planning
	*Free text *
	advance* near (care plan* or health care plan* or healthcare plan* or medical plan* or treatment plan* or directiv* or care directiv* or health care directiv* or healthcare directive* or treatment directiv* or care wish* or treatment wish*):ti,ab,kw (Word variations have been searched)
	end of life near (decision* or communicat* or care communicat* or discussion* or plan* or care plan* or wish* or conversation)
	plan* for the end of life or plan* for end of life
	((advance near/3 plan*) or ((living or patient) near/2 (will* or contract* or decision* or participat*)) or (advance near/1 directive*) or (Attorney near/2 Power) or (psychiatric near/1 will*))
	Search terms nursing home:
	*MESH terms*
	Nursing Homes
	Long-Term Care
	*Free text*
	(nursing home* or care home* or long-term care or longterm care or old peoples home* or rest* home* or home* for the aged or intermediate care facilit* or skilled nursing facilit*)
	nursing home* or “home* for the aged” or hospice*)
	Search terms patient group – dementia:
	*Free text*
	(dement* or alzheimer* or “Frontotemporal lobar degeneration” or “Lewy Body disease”):ti,ab,kw (Word variations have been searched)

## Abbreviations

ACP: advance care planning
AD: advance directives
EoLC: end-of-life care
GSFCH: gold standards framework for care homes
MAPP: making advance care planning a priority
MMSE: mini mental status examination
NH: nursing home
PICO: problem/population, intervention, comparison and outcomes
POLST: physician orders for life-sustaining treatment
